# Induction therapy with high‐dose fluconazole plus flucytosine for human immunodeficiency virus‐uninfected cryptococcal meningitis patients: Feasible or not?

**DOI:** 10.1111/myc.13528

**Published:** 2022-09-26

**Authors:** Hua‐Zhen Zhao, Jia‐Hui Cheng, Ling‐Hong Zhou, Yu Luo, Rong‐Sheng Zhu, Ying‐Kui Jiang, Xuan Wang, Li‐Ping Zhu

**Affiliations:** ^1^ Shanghai Key Laboratory of Infectious Diseases and Biosafety Emergency Response, National Medical Center for Infectious Diseases, Department of Infectious Diseases Huashan Hospital, Fudan University Shanghai China

**Keywords:** cryptococcal meningitis, fluconazole, high dose, HIV‐uninfected, induction therapy

## Abstract

**Background:**

Cryptococcal meningitis (CM) is increasingly recognised in human immunodeficiency virus (HIV)‐uninfected patients with high mortality. The efficacy and safety profiles of induction therapy with high‐dose fluconazole plus flucytosine remain unclear.

**Methods:**

HIV‐uninfected CM patients who received high‐dose fluconazole (800 mg/d) for initial therapy in Huashan Hospital were included in this retrospective study from January 2013 to December 2018. Efficacy and safety of initial therapy, clinical outcomes and risk factors were evaluated.

**Results:**

Twenty‐seven (71.1%) patients who received high‐dose fluconazole with flucytosine combination therapy and 11 (28.9%) having fluconazole alone for induction therapy were included. With a median duration of 42 days (IQR, 28–86), the successful response rate of initial therapy was 76.3% (29/38), while adverse drug reactions occurred in 14 patients (36.8%). The rate of persistently positive cerebrospinal fluid (CSF) culture results was 30.6% at 2 weeks, which was significantly associated with CSF CrAg titre >1:1280 (OR 9.56; 95% CI 1.40–103.65; *p* = .010) and CSF culture of *Cryptococcus* >3.9 log_10_CFU/ml (OR 19.20; 95% CI 1.60–920.54; *p* = .011), and decreased to 8.6% at 4 weeks. One‐year mortality was 15.8% (6/38), and low serum albumin (35 g/L) was found as an independent risk factor for 1‐year mortality (HR 6.31; 95% CI 1.150–34.632; *p* = .034).

**Conclusions:**

Induction therapy with high‐dose fluconazole (800 mg/d), combined with flucytosine, effectively treated HIV‐uninfected CM and was well tolerated. Long‐term fluconazole treatment with continued monitoring is beneficial for patients with persistent infection.

## 1 INTRODUCTION

Cryptococcal meningitis (CM) is the most prevalent fungal infection of the central nervous system with a significant burden in both human immunodeficiency virus (HIV)‐infected and HIV‐uninfected population.^1^, ^2^ With an estimated incident cases of 223,100 annually, CM has been one of the leading causes of AIDS‐related deaths worldwide.^3^ In HIV‐uninfected population, an increasing amount of cryptococcosis was recognised in patients with predisposing factors including solid organ transplants, haematological diseases, liver cirrhosis, immunosuppressant therapies or even those with apparently normal immune status.^4^, ^5^, ^6^ Notably, CM was diagnosed mainly in HIV‐uninfected population in China, accounting for twice as many as HIV‐infected cases.^7^ The persistent burden as well as poor outcome in these patients remains a challenge to public health.

Effective antifungal treatment regimens that can be safely administrated are a key to favourable outcome. Although amphotericin B combined with flucytosine was the recommended induction therapy against HIV‐uninfected CM,^8^ its use was frequently limited by significant toxicity including anaemia, kidney and liver impairment, and electrolyte abnormalities.^9^ Meanwhile, fluconazole has been widely used in consolidation and maintenance therapy with excellent tolerability. Its safety profile and availability make it an attractive alternative initial therapy to amphotericin B‐based regimens. However, as an induction therapy, high‐dose fluconazole (800 mg/d) for HIV‐uninfected CM has not been well described. Therefore, we performed this retrospective study to evaluate the efficacy, safety and outcome of high‐dose fluconazole (800 mg/d), alone or combined with flucytosine, initially treating HIV‐uninfected CM patients.

## 2 PATIENTS AND METHODS

### 2.1 Study Design

This retrospective study was performed among the HIV‐uninfected CM patients who were admitted to Huashan Hospital, Fudan University between January 2013 and December 2018. Patients diagnosed with proven CM and receiving high‐dose (800 mg/d) fluconazole, alone or combined with flucytosine, as initial therapy were included. Patients with HIV infection were excluded. Medical data were retrieved from the electronic medical record system of Huashan Hospital, which included baseline demographics, medical histories, radiological and laboratory findings, treatments and clinical outcomes. This study was reviewed and approved by the Institutional Review Board of Huashan Hospital.

### 2.2 Definition

A diagnosis of CM was confirmed when any of the following criteria were met: (1) positive culture of *Cryptococcus* from cerebrospinal fluid (CSF); (2) positive CSF ink smear; (3) compatible histopathological findings of *Cryptococcus* in brain tissue or (4) positive cryptococcal antigen (CrAg) lateral flow assay test (IMMY, Norman, OK, USA) in CSF. Pulmonary cryptococcosis is diagnosed according to our previous definition.^5^ Initial therapy was defined when an antifungal regimen was initially administered for at least 7 consecutive days.

### 2.3 Analysis of Cryptococcus Isolates

Fresh CSF samples were analysed for quantitative fungal culture with “St. George's Method.”^10^ Phenotypic characterisation of clinical isolates was identified by chemotyping on canavanine‐glycine‐bromothymol blue (CGB) medium. *Cryptococcus* species and sequence types were characterised by multilocus sequence typing (MLST).^11^ In vitro susceptibility of *Cryptococcus* isolates to fluconazole was performed using the broth microdilution method by the Clinical and Laboratory Standards Institute M27 (4th edition) protocol.^12^ *Candida parapsilosis* ATCC 22019 and *Candida krusei* ATCC 6258 were used as quality control (QC) isolates. Minimal inhibitory concentration (MIC) was read as the lowest concentration which inhibited growth by 50% compared to that of the control. Isolates were considered as susceptible when MIC exhibited ≤8 mg/L.

### 2.4 Fluconazole Concentration and Analysis

Blood and CSF samples were collected at least 5 days following the initial dose when steady‐state concentrations of fluconazole were reached. Samples were obtained before the first fluconazole dose in the morning and stored at −20°C before analysis. Fluconazole concentrations were measured using a validated liquid chromatography–tandem mass spectrometry assay. The concentration‐time area under the curve (AUC) of both serum and CSF was calculated using fluconazole concentrations multiplied by 24 h as described in previous studies.^13^ Proportions of patients achieving therapeutic concentrations with AUC/MIC ratio of >100 were calculated to evaluate fluconazole's mycologic activity.

### 2.5 Efficacy and Safety

Clinical responses were evaluated as success (complete response [CR] or partial response [PR]) or failure (stable response, progression of disease, or death) according to previous criteria at 2 time points: the end of initial therapy and 10 weeks after the initiation of antifungal therapy.^14^ Also, survival along with neurological sequelae was analysed at 1‐year follow‐up.

Adverse drug reactions (ADRs) were monitored throughout the administration of high‐dose fluconazole. The severity for each ADR was reported as Grade 1 through 5 in a sequence of ascending degrees in accordance with Common Terminology Criteria for Adverse Events, version 5.0.^15^ The relationships of ADRs and high‐dose fluconazole were described as certain, probable and possible based on the World Health Organization‐Uppsala Monitoring Center system for standardised case causality assessment.^16^

### 2.6 Statistical Analysis

Continuous variables were compared with *t*‐test or the non‐parametric Mann–Whitney test. Proportions were compared with Fisher's exact test. Relationships between characteristics and 1‐year survival were examined with Kaplan–Meier analysis, with the prognostic indicators compared by the log‐rank test. The Cox proportional hazards model was used to assess the effects of potentially confounding variables on the risk of mortality. A Spearman correlation coefficient was calculated to examine the relationship between fluconazole concentration in serum and CSF. A *p*‐value of <.05 was considered statistically significant.

## 3 RESULTS

### 3.1 Demographics and Clinical Manifestations

Thirty‐eight HIV‐uninfected patients with proven CM were included in the study. Of them, 17 (44.7%) patients had multi‐site cryptococcosis involving lung (*n* = 11 [28.9%]) and bloodstream (*n* = 8 [21.1%]). CM was diagnosed at a median of 23 days (IQR, 14.5–40) after the onset of symptoms. As shown in Table 1, 78.9% were male patients, and the median age was 49.5 years. Patients with predisposing factors accounted for 60.5%, 14 of whom suffered more than 1 underlying conditions. Headache (89.5%) and fever (73.7%) were the most common symptoms. Altered mental status was observed in six patients, including three mild and three moderate cases according to Glasgow Coma Scale.

**TABLE 1:** Baseline characteristics and manifestations of 38 patients treated with high‐dose fluconazole as initial therapy

Variables	*N* = 38 (%)
Male	30 (78.9)
Age, median (IQR)	49.5 (41.3–62.0)
Underlying conditions	23 (60.5)
Corticosteroids use	12 (31.6)
Type 2 diabetes mellitus	9 (23.7)
Liver cirrhosis	3 (7.9)
Nephrotic syndrome	3 (7.9)
Kidney transplantation	2 (5.3)
Chronic nephritis	2 (5.3)
Pemphigus	2 (5.3)
ITP	1 (2.6)
SLE	1 (2.6)
MG	1 (2.6)
Monoclonal gammopathy	1 (2.6)
Idiopathic CD4 lymphocytopenia	1 (2.6)
Gastric cancer	1 (2.6)
Symptoms and signs	
Headache	34 (89.5)
Fever	28 (73.7)
Vomiting	20 (52.6)
Meningeal irritation	14 (36.8)
Dizziness	8 (21.1)
Altered mental status	6 (15.8)
Seizure	1 (2.6)
Cranial nerve defect	22 (57.9)
Visual loss	5 (13.2)
Diplopia	2 (5.26)
Visual field defect	9 (23.7)
Downgrade of visual sensitivity	6 (15.8)
Hearing deficit	13 (34.2)
Cranial nerve III dysfunction	1 (2.6)
Cranial nerve VI dysfunction	2 (5.3)
Baseline cranial radiology^a^	
Abnormal imaging	32 (94.1)
Parenchyma lesions	27 (79.4)
Frontal lobe	21 (61.8)
Parietal lobe	16 (47.1)
Periventricular region	13 (38.2)
Ventricular enlargements	10 (29.4)
Meningeal enhancements^b^	14 (56.0)
Ring‐enhancing cerebral lesions^b^	3 (12.0)

*Note*: Data are presented as No. (%) unless otherwise indicated.

Abbreviations: CrAg, cryptococcal antigen; CSF, cerebrospinal fluid; MG, myasthenia gravis; ITP, idiopathic thrombocytopenic purpura; IQR, interquartile range; SLE, systemic lupus erythematosus.

^a^ A total of 34 patients underwent cranial MR or CT scan at baseline.

^b^ A total of 25 patients had contrast‐enhanced MRI images at baseline.

Abnormal results of cranial MR or CT scan were shown in 94.1% (32/34) of the patients. Parenchyma lesions (27/34, 79.4%) were most commonly seen, mainly presented as focal ischemia (24/34, 70.6%) and localised frequently in the frontal lobe (21/34, 61.8%). Besides, ventricular enlargements accounted for 29.4% (10/34). Among the 25 patients who had contrast‐enhanced MRI, 56% (14/25) had meningeal enhancements and 12% (3/25) had ring‐enhancing cerebral lesions (Table 1).

### 3.2 Laboratory Examinations

Pretreatment CSF examinations were performed in all 38 patients including 36 samples from lumbar punctures, one from continuous lumbar CSF drainage and one from Ommaya reservoir. Most patients (25/35, 71.4%) had elevated opening pressure of lumbar puncture. CSF examinations showed elevated white blood cell count (33/38, 86.8%) and protein level (36/38, 94.7%) and decreased glucose concentration (28/38, 73.7%). CSF CrAg was positive in all 37 patients with documents with a median titre of 1:1280. Positive culture of *Cryptococcus* and positive ink smear of CSF were found in 36 (94.7%) and 30 (78.9%) patients, respectively. Quantitative fungal culture showed a median of 3.90 log_10_CFU/ml (IQR, 2.11–4.48).

A total of 35 *Cryptococcus* isolates were performed with strain identification and susceptibility test to fluconazole. Three were identified as *C. gattii* by CGB culture. MLST analysis showed VNI‐ST5 (29/35, 82.9%), VNI‐ST63 (1/35, 2.9%), VNIV‐ST109 (1/35, 2.9%), one unclassified VN isolates and three unclassified VG isolates. For drug susceptibility test, the MIC values of QC strain ATCC 22019 and ATCC 6258 in this study were 2 and 32 mg/L, respectively. All *C. neoformans* isolates exhibited susceptible with MICs ranging from 2 to 8 mg/L, an MIC_50_ of 4 mg/L, an MIC_90_ of 8 mg/L, modes of 4 mg/L and a geometric mean of 4.97 mg/L, respectively. MICs of *C. gattii* isolates were 4 mg/L in one and 16 mg/L in two patients.

For fluconazole concentration measurements, serum samples of 18 patients were collected on a median of 29 (IQR, 20–40) days following the initiation of high‐dose fluconazole, presenting a median level of 34.51 μg/ml (IQR, 27.65–39.38). CSF measurements were carried out on 23 patients on a median of 29 days (IQR, 8.5–27.0). The median level in CSF was 30.82 μg/ml (IQR, 23.98–40.50). Ratios of CSF/serum fluconazole concentration showed a median of 0.70 (IQR, 0.58–1.00), and no significant correlation was revealed between CSF and serum levels (*p* = .491; *r* = .193).

### 3.3 Efficacy and Safety

The median duration of initial therapy for all 38 patients was 42 days (IQR, 28–86). Of them, the combination of high‐dose fluconazole and flucytosine (78 – 100 mg/kg/d) was administered in 27 patients for a median of 41 days (IQR, 28.5–105.5). High‐dose fluconazole monotherapy (median duration, 43 days [IQR, 15.5–72]) was used in the remaining 11 patients, including four with chronic kidney diseases, two with cytopenia caused by haematological diseases, two with liver cirrhosis, one with gastric carcinoma, and two with no underlying conditions.

**FIGURE 1:**
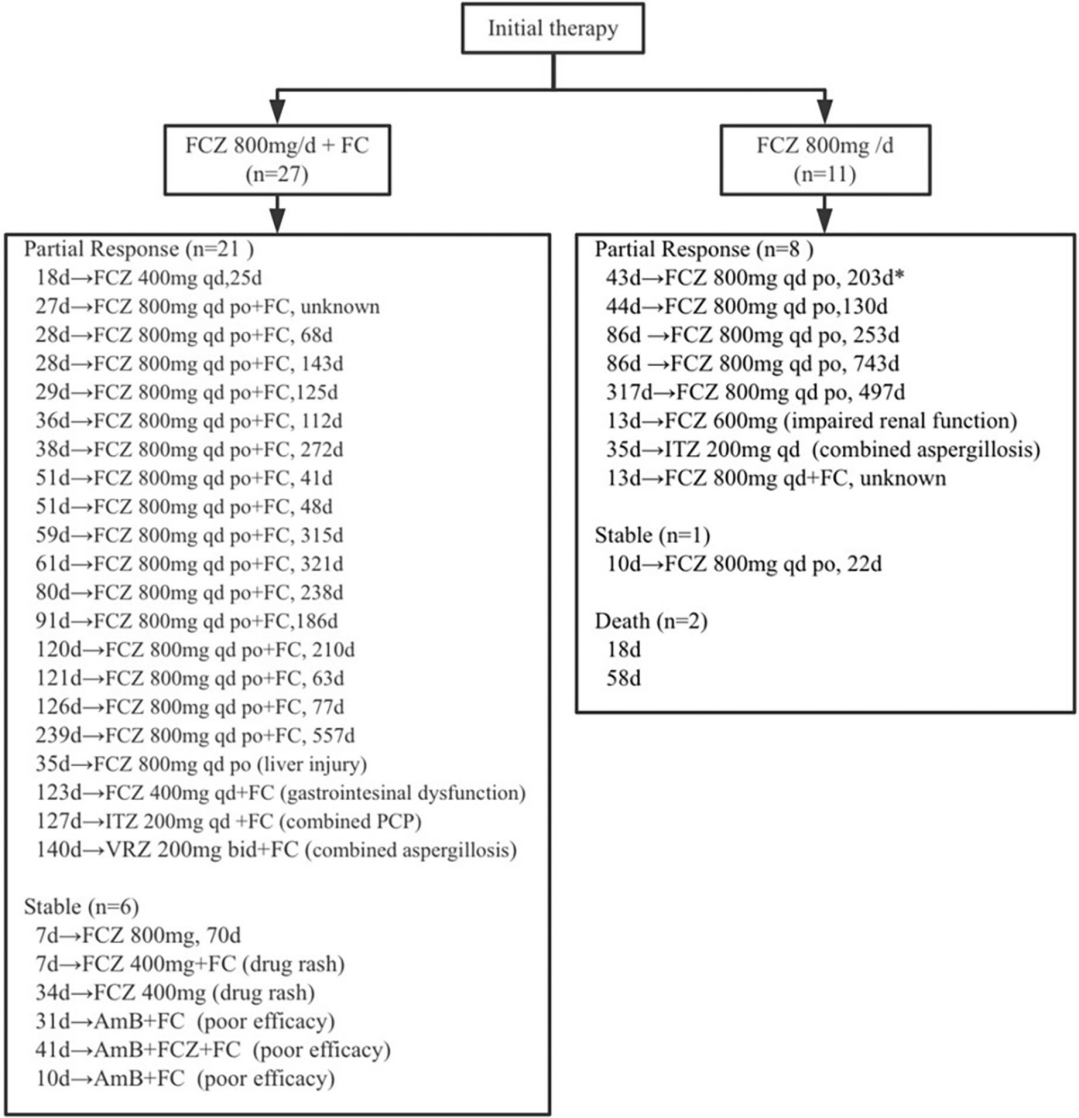
Profile of therapeutic process of 38 patients treated with high‐dose fluconazole‐based initial therapy. An example for reading the figure: *means that the patient received intravenous fluconazole 800 mg/d for 43 days as induction therapy and achieved partial response, then followed by consolidation therapy with oral fluconazole 800 mg/d for 203 days. Abbreviations: AmB, amphotericin B; FC, flucytosine; FCZ, fluconazole; ITZ, itraconazole; VRZ, voriconazole

The successful response rate of initial therapy was 76.3% (29/38; all had PR). Seven (18.4%) patients remained stable response, and two (5.3%) died at initial therapy (one died of septic shock caused by *E.coli*, the other of brain hernia) (Figure 1). The successful response rates were similar between patients receiving combination therapy and monotherapy (77.8% vs 72.7%; *p* = 1.000). At 10 weeks, the total successful response rate reached 86.8% (CR in three patients, PR in 30), while two patients remained stable, and three patients died.

A total of 29 ADRs occurred in 14 patients (36.8%) during initial therapy, including liver dysfunction, hypokalemia, anaemia, rash and gastrointestinal symptoms (Table 2). Ten patients (26.3%) developed Grade III or IV ADRs, which accounted for 41.4% (12/29) of all episodes. Most ADRs (28/29, 96.6%) were possibly related to initial regimens, and the remaining 1 drug rash was in certain causality. Three patients endured fluconazole dosage reduction due to ADRs (2 with rash, and 1 with gastrointestinal dysfunction). No significant difference was discovered between two treatment groups in total ADRs (10/27 vs. 4/11, *p* = 1.000) or Grade III‐IV ADRs (7/27 vs. 3/11, *p* = 1.000).

**TABLE 2:** Adverse events occurred in initial therapy per treatment group

Type/Grade of event	FCZ monotherapy (*n* = 11)		FCZ + FC (*n* = 27)	
	Grade I‐II	Grade III‐IV	Grade I‐II	Grade III‐IV
Gastrointestinal symptoms	1(9.1)	0 (0)	3(11.1)	0 (0)
Rash	0 (0)	0 (0)	1(3.7)	2 (7.4)
Anaemia	0 (0)	0 (0)	1(3.7)	0 (0)
Elevated ALT	0 (0)	0 (0)	1(3.7)	1 (3.7)
Elevated AST	1 (9.1)	0 (0)	1(3.7)	1 (3.7)
Elevated ALP	2 (18.2)	0 (0)	2(7.4)	0 (0)
Elevated GGT	0 (0)	1 (9.1)	2(7.4)	2 (7.4)
Elevated bilirubin	0 (0)	0 (0)	1(3.7)	0 (0)
Hypokalemia	0 (0)	2(18.2)	1(3.7)	3 (11.1)
Total	4	3	13	9

*Note*: Data are presented as No. (%) unless otherwise indicated.

Abbreviations: ALT, alanine aminotransferase; AST, aspartate aminotransferase; ALP, alkaline phosphatase; FC, flucytosine; FCZ, fluconazole; GGT, gamma‐glutamyltransferase.

### 3.4 Persistent Infection and Prognosis

At 2 weeks after the initiation of high‐dose fluconazole, CSF sterilisation was achieved in 25 patients (69.4%) of the 36 patients who had positive culture of *Cryptococcus* at baseline. At 4 weeks, CSF culture remained positive in 3 patients (3/35, 8.6%). Factors potentially associated with results of CSF culture at 2 and 4 weeks were included in univariate analysis (Table 3). Higher risks of positive CSF culture at 2 weeks were revealed in patients with CSF CrAg titre >1:1280 (OR 9.56; 95% CI 1.40–103.65; *p* = .010) and CSF culture of *Cryptococcus* >3.9 log_10_CFU/ml at baseline (OR 19.20, 95% CI 1.60–920.54; *p* = .011). Neither susceptibility to fluconazole nor fluconazole exposure exhibited a significant difference between these two groups. The 3 patients with persistently positive culture results at 4 weeks all had high CSF CrAg titres (>1:1280) and high quantities of *Cryptococcus* (>3.9 log_10_CFU/ml). Two of them (67.7%) continued to receive high‐dose fluconazole, achieved PR after initial therapy and survived at 1‐year follow‐up. The remaining one patient with persistent infection at 4 weeks died of respiratory failure at 55 days. Proportion of patients receiving fluconazole plus flucytosine was higher in group with sterile CSF both at 2 weeks (19/25 vs. 8/11, *p* = 1.000) and 4 weeks (25/32 vs. 2/3, *p* = 0.553) (Table 3).

**TABLE 3:** Factors associated with persistently positive culture of CSF at 2 and 4 weeks

Factors	Negative CSF culture at 2 weeks (*n* = 25)	Positive CSF culture at 2 weeks (*n* = 11)	Negative CSF culture at 4 weeks (*n* = 32)	Positive CSF culture at 4 weeks (*n* = 3)	*p* (2 weeks)	*p* (4 weeks)
Male	21/25	7/11	26/32	2/3	.214	.499
Age > 60 years	9/25	1/11	9/32	1/3	.127	1.000
Predisposing factors	15/25	7/11	19/32	2/3	1.000	1.000
Time to diagnose >30 days	8/25	5/11	11/32	2/3	.475	.541
GCS score < 15	4/25	2/11	6/32	0/3	1.000	1.000
Radiological findings						
Meningeal enhancement	8/17	5/7	12/22	1/2	.386	1.000
Ventricular enlargements	8/22	2/10	9/28	1/3	.440	1.000
Baseline laboratory examinations						
Serum creatinine, median (IQR)	63.0 (42.8–82.0)	58.0 (52.0–68.0)	61.0 (45.0–73.0)	56.0 (48.0–61.0)	.749	.504
eGFR<50	2/25	1/11	2/32	0/3	1.000	1.000
Opening pressure of lumbar puncture >300mmH_2_O	3/22	4/11	5/27	1/3	.186	.501
CSF glucose <2.5 mmol/L	17/25	10/11	23/32	3/3	.223	.553
CSF protein >600 mg/L	23/25	9/11	28/32	3/3	.570	1.000
CSF CrAg titre >1:1280	8/25	9/11	13/32	3/3	.010	.086
Serum CrAg titre>1:1280	8/22	6/11	11/29	2/3	.459	.558
Log_10_CFU/ml >3.9 (median)	5/17	8/9	9/22	3/3	.011	.096
Clinical pharmacology						
*Cryptococcus neoformans*	21/23	10/11	27/30	3/3	1.000	1.000
*Cryptococcus* Susceptible to FCZ	22/23	10/11	28/30	3/3	1.000	1.000
FCZ concentrations in CSF >30.82 μg/ml (median)	6/15	5/8	8/20	3/3	.400	.093
FCZ concentrations in serum >34.51 μg/ml (median)	7/13	2/5	9/17	0/1	1.000	1.000
AUC_serum_/MIC >100	9/12	5/5	13/16	1/1	.515	1.000
AUC_csf_/MIC >100	11/15	6/8	14/20	3/3	1.000	.539
Treatment and outcome						
Combination therapy with FCZ and FC	19/25	8/11	25/32	2/3	1.000	.553
Successful response to initial therapy	18/25	9/11	25/32	2/3	.690	.553
Successful response at 10 week	22/25	9/11	29/32	2/3	.631	.313
Survival at 1 year	21/25	9/11	28/32	2/3	1.000	.380

Abbreviations: AUC, area under the curve; CFU, colony‐forming unit; CSF, cerebrospinal fluid; CrAg, cryptococcal antigen; eGFR, estimated glomerular filtration rate; FC, flucytosine; FCZ, fluconazole; GCS, Glasgow coma scale; IQR, interquartile range; MIC, minimal inhibitory concentration.

**FIGURE 2:**
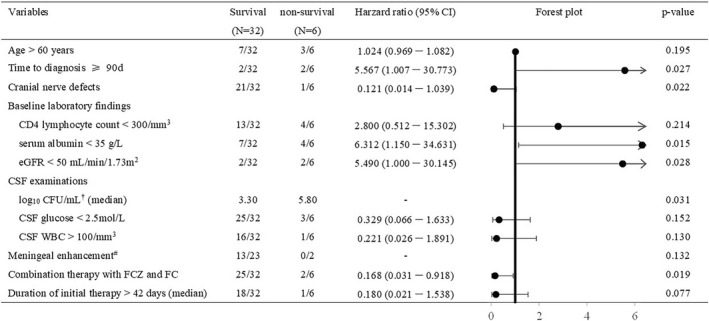
Univariate analysis of factors influencing 1‐year mortality among 38 CM patients. Data are presented as No. (%) unless otherwise indicated; † Data of CFU were available in 26 patients. # Data of meningeal enhancements were available in 25 patients. CI, confidence interval; CSF, cerebral spinal fluid; CFU, colony forming unit; eGFR, estimated glomerular filtration rate; FC, flucytosine; FCZ, fluconazole.

All‐cause 1‐year mortality rate was 15.8% (6/38), and detailed information about the 6 non‐survivors is listed in Table 4. In univariate analysis, time to diagnosis >90 days, less cranial nerve defect, serum albumin <35 g/L, eGFR <50 ml/min/1.73m^2^, high CSF fungal burden and initial therapy with fluconazole monotherapy were associated with higher 1‐year mortality (Figure 2). In multivariate analysis, independent risk factor associated with 1‐year mortality was low serum albumin (< 35 g/L; HR 6.31, 95%CI 1.150–34.632; *p* = .034). Among the 32 survivors, three (9.39%) presented sequelae related to CM, including visual loss (*n* = 2), hearing loss (*n* = 2) and incontinence (*n* = 1). No relapse occurred in 32 survivors.

**TABLE 4:** Patients died within 1‐year follow‐up

Patient	Sex	Age	days to diagnosis	Predisposing factors	GCS score	Isolate	Initial therapy	Initial response	Death	Causality
1	F	44	29	Nephritis, GC	15	VNI‐ST5	FCZ,17d	death	20d	Septic shock
2	M	62	182	Monoclonal gammopathy	15	ND	FCZ,10d	stable	32d	Haematological disease
3	M	78	16	MG, GC	15	VNI‐ST5	FCZ + FC,34d	stable	55d	Respiratory failure
4	M	74	886	Gastric cancer	15	VG‐unclassified	FCZ,58d	death	58d	Brain hernia
5	M	32	6	SOT, GC	15	VNI‐ST5	FCZ,35d	PR	83d	Respiratory failure
6	F	41	19	None	15	VNI‐ST5	FCZ + FC,35d	PR	104d	sepsis

Abbreviations: F, female; FC, flucytosine; FCZ, fluconazole; GC, glucocorticoid; M, male; ND, not detected; PR, partial response; SOT, solid organ transplantation.

## 4 DISCUSSION

Amphotericin B is the mainstay of therapy for HIV‐infected CM due to its superior efficacy, though its severe adverse events greatly restrict its clinical use. Fluconazole as a safe and well‐tolerated anti‐fungal choice has shown high penetration of blood–brain barrier, but still the alternative choice for HIV‐infected CM.^8^ Recent clinical trials showed that fluconazole (800 – 1200 mg/d) along with flucytosine was a good choice for initial therapy in resource‐limited areas, the 10‐week survival rates of which were 57% to 87%, and higher doses of fluconazole (up to 2000 mg/d) were associated with higher survival.^17^, ^18^ Thus, high‐dose fluconazole (800 – 2000 mg/d) combined with flucytosine, as an alternative initial therapy for HIV‐infected CM, was recommended by cryptococcosis guidelines updated in 2010, especially for patients with low tolerance to the substantial ADRs of amphotericin B.^8^ Later, a randomised controlled trial involving 721 HIV‐infected CM further proved the non‐inferior efficacy and better safety of high‐dose fluconazole (1200 mg/d) plus flucytosine compared with 2 weeks of amphotericin B induction treatment.^9^ Correspondingly in the 2018 guidelines for cryptococcosis in HIV‐infected patients by World Health Organization, 1 week of fluconazole (1200 mg) following 1 week of amphotericin B‐based therapy was promoted to be the first‐line antifungal regimen for the induction therapy.^19^ This study provides a comprehensive analysis of HIV‐uninfected CM who initially received high‐dose fluconazole (800 mg/d) mostly with flucytosine as antifungal treatment. Our data demonstrated that fluconazole could be administered as initial antifungal treatment, which exhibited favourable efficacy and safety for HIV‐uninfected CM patients.

When initially treated HIV‐uninfected CM patients with fluconazole (200 – 439 mg/d), early studies exhibited varying successful response rates from 67% to 89%.^20^, ^21^, ^22^, ^23^ However, PK/PD analysis showed that low‐dose fluconazole (400 mg/d) resulted in underexposure in 21% and 23% of patients evaluating early and late target attainment, respectively.^24^ Higher dose fluconazole was gradually applied in HIV‐uninfected patients in clinical treatment. A recent retrospective study suggested that fluconazole (400 – 800 mg/d) with flucytosine led to no significant difference in fungal clearance (74.4% vs 70.2%, *p* = .814) and 12‐week response (69.7% vs 72.3%, *p* = .820) compared with regimen of amphotericin B combined with flucytosine.^25^ Furthermore, we once conducted a retrospective study in HIV‐uninfected patients with CM who were either refractory or intolerant to previous antifungal treatment. High‐dose fluconazole (800 mg/d) with or without flucytosine was administered for salvage therapy, showing favourable results with a successful response rate of 83.7% and no grade 3 or 4 ADRs.^10^ In our current study, underlying diseases including liver cirrhosis, nephrotic syndrome, nephritis, kidney transplantation, anaemia, leukopenia and thrombocytopenia were diagnosed in as many as 23 (60.5%) patients. A markedly high proportion of patients (76.3%) exhibited a successful response to high‐dose fluconazole. Meanwhile, ADRs observed in our study occurred in 36.8% of the 38 patients, and over a half (58.6%) of all episodes were mild to moderate in severity evaluation, which was in consistence with the well tolerance of high‐dose fluconazole (800‐2000 mg/d) documented in previous study.^17^, ^18^ Although neutropenia and thrombocytopenia have been reported to occur more frequently when flucytosine was added compared with fluconazole monotherapy,^26^ we observed no such ADR in these 38 patients, and the incidence was as low as 2.3% in salvage therapy with high‐dose fluconazole.^10^ To sum up, high‐dose fluconazole (800 mg/d) unprecedentedly showed non‐inferior efficacy to amphotericin B in HIV‐uninfected patients with CM, while having more favourable safety profiles whether combined with flucytosine or not.

One of the important factors for the leading situation of amphotericin B is that it shows excellent fungicides effect for *Cryptococcus* rather than inhibitor agent such as fluconazole. It is noteworthy that persistent infection did exist during fluconazole administration, with CSF sterilisation rates of 69.4% and 91.4% at 2 weeks and 4 weeks, respectively. Independent predictors reported to be associated with persistent infection included high fungal burden representing as high CrAg titres and positive extraneural cultures, poor nutrition status and insufficient doses of antifungal drugs.^27^, ^28^ Likewise, we also revealed that high baseline CSF CrAg titre (>1:1280) and high quantity of *Cryptococcus* (>3.9 log_10_CFU/ml) were associated with a 9.56‐ and 19.20‐fold increased risk of positive CSF culture at 2 weeks, respectively. The fungistatic effect of fluconazole on *Cryptococcus* could also contribute to persistent infection. However, previous studies in HIV‐associated CM showed 2‐week fungal clearance rates of 25.5%–59.7% in patients treated with amphotericin B, and data in HIV‐uninfected patients demonstrated no significant difference of persistent infection between groups receiving amphotericin B and fluconazole (sterility rate at 4 weeks: 55.8% vs 40.4%, *p* = .191).^25^, ^27^, ^28^, ^29^

Recent years, it has been reported that the fluconazole‐resistant *Cryptococcus* has been increasing. Proportions of non‐susceptible isolates of *C. neoformans* (MIC >8 mg/L) varied from 0%–11.1% among different regions of China and 1.6%–3.0% worldwide.^30^, ^31^, ^32^, ^33^, ^34^, ^35^Our analysis further revealed that neither susceptibility to fluconazole, drug concentration nor exposure had significant association with CSF sterilisation. Similarly, O'Connor et al investigated MICs to fluconazole of *Cryptococcus* isolates obtained from 88 patients before they received induction therapy, demonstrating that there was no relationship between MICs to fluconazole and survival or fungal clearance.^36^ Moreover, there is a theoretical speculation that *Cryptococcus* displays the phenomenon of heteroresistance under azole exposure, whereby an intrinsic or acquired resistant sub‐population can emerge from within a predominantly susceptible single strain.^37^, ^38^ Although the mechanism remained poorly understood and may be associated with drug efflux level of isolates, heteroresistance was observed to be successfully suppressed when treated with combination therapy of fluconazole and flucytosine.^39^ As a partner drug with fluconazole, flucytosine in our study slightly increased efficacy, accelerated CSF sterilisation and significantly improved 1‐year survival in univariant analysis (*p* = .019) compared with fluconazole monotherapy. Similar results were shown in a recent national program in South Africa that flucytosine‐containing regimen was associated with a 53% reduction in mortality of CM (*p* < .0001).^40^ Most notably, despite of the existence of persistent infection in our study, it made no difference on 1‐year survival. High‐dose fluconazole was continued in the 11 patients who had nonsterile CSF at 2 weeks, and patients with positive fungal cultures decreased to 3 at 4 weeks, 2 of whom even survived at 1‐year follow‐up. Taken together, these results showed us the necessity of long‐term as well as high‐dose fluconazole administration, especially for patients who had high fungal load at baseline and no indication of deterioration during initial treatment.

This study had some limitations. Since it is a single‐centre study with limited number of patients included, conclusions about induction therapy with high‐dose fluconazole regimens should be interpreted with caution. Bias existed due to the retrospective nature of this study also reduced the universality. Moreover, *Cryptococcus* isolates during follow‐up were not preserved, which limited the dynamic monitoring of drug susceptibility under fluconazole exposure. Thus, larger‐scale randomised controlled trials are necessary to better evaluate the efficacy and safety of high‐dose fluconazole for initial therapy in HIV‐uninfected CM in the future.

Our study suggested that high‐dose fluconazole (800 mg/d) plus flucytosine effectively treated HIV‐uninfected CM and was well tolerated as induction therapy. Persistent infection was associated with high fungal burden. Long‐term administration with continued monitoring is recommended especially for patients with persistent infection.

## AUTHOR CONTRIBUTIONS

**Hua‐Zhen Zhao**: Writing – Original Draft Preparation; Data Curation; Formal Analysis. **Jia‐Hui Cheng**: Writing – Original Draft Preparation; Investigation; Methodology. **Ling‐Hong Zhou**: Data Curation; Investigation; Methodology. **Yu Luo**: Methodology. **Rong‐Sheng Zhu**: Visualisation. **Ying‐Kui Jiang**: Writing – Review & Editing. **Xuan Wang**: Resources. **Li‐Ping Zhu**: Conceptualisation; Resources; Funding Acquisition; Writing – Review & Editing.

## CONFLICT OF INTEREST

The authors have no conflicts of interest to declare.

## Data Availability

The data that support the findings of this study are available from the corresponding author upon reasonable request.
